# Supplementary material for: Multisite Metagenomic Next-Generation Sequencing Improved Diagnostic Performance for Sepsis-Associated Lymphopenia Patients
Source: Microbiol Spectr. 2022 Dec 1;10(6):e03532-22. doi: 10.1128/spectrum.03532-22 (PMC9769823; doi:10.1128/spectrum.03532-22)
Supplement: Supplemental file 1 — Supplemental material. Download spectrum.03532-22-s0001.pdf, PDF file, 0.4 MB [file spectrum.03532-22-s0001.pdf]

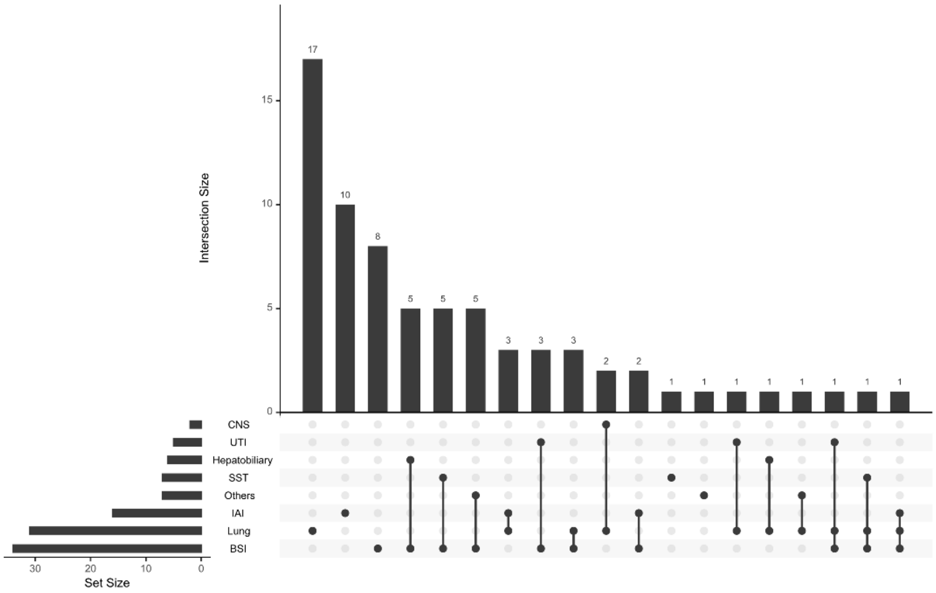

Supplement Figure 1. **Venn Upset plot of the distribution of clinically suspeced infection sites**; Each row represents the site of infection; the number at the end of each bar represents the number of cases with this infection site; and each column represents one group of patients with multiple sites of infection, which are represented by the black dots' location and connecting lines. mNGS, metagenomic next-generation sequencing. CMT, conventional microbiological test.

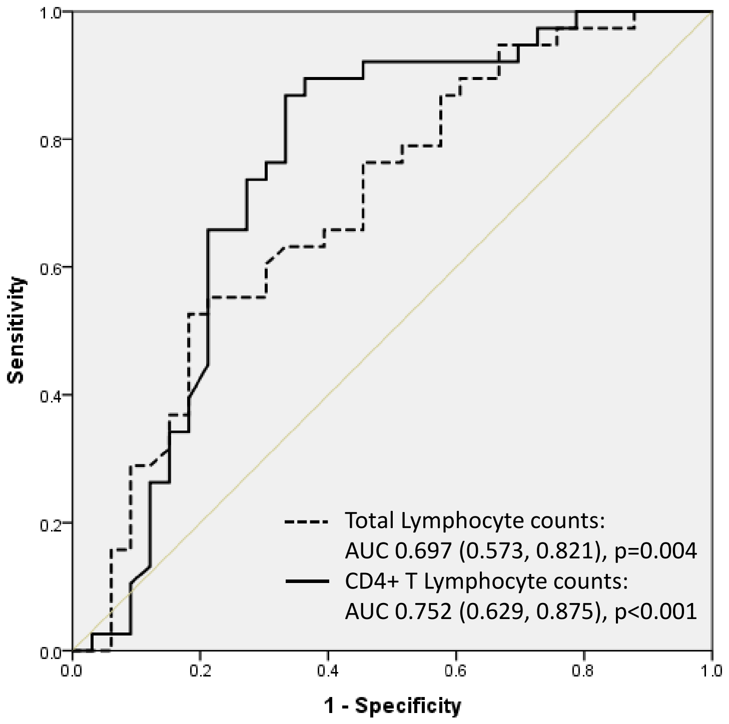

Supplemental Figure 2. Receiver operating characteristic analysis of total and CD4+ T lymphocyte counts in predicting matching results between plasma mNGS detection and CMT analysis of clinically suspected infection sites

mNGS, metagenomic next-generation sequencing. CMT, conventional microbiological test.

Supplement Table 1. Demographic and clinical characteristics of study patients

	**Match between plasma mNGS and CMT on infection sites**		
	**Unmatched**	**Complete or partially matched**	**P**
**N**	33	38	
**Age**	59.1±17.1	60.6±16.7	0.711
**Sex，male**	19 (57.6%)	27 (71.1%)	0.236
**APACHE II**	22.6±10.6	21.2±9.8	0.568
**SOFA**	4.9±3.5	6.1±4.6	0.213
**WBC, cells/mm3**	13.5±7.1	11.1±6.5	0.140
**Lymphocytes, cells/mm3**	761±359	548±247	**0.005**
**B lymphocyte**	124±103	91±60	0.095
**T lymphocyte**	577±317	395±207	**0.005**
**CD4+ T**	333±199	230±120	**0.009**
**CD8+ T**	199±147	148±114	0.103

**NK cell**	58±55	84±122	0.253
**C3, g/L**	0.84±0.33	0.75±0.26	0.287
**C4, g/L**	0.18±0.11	0.17±0.06	0.441
**IL-6**	178.2±287.5	165.2±271.8	0.876
**IL-8**	279.8±614.7	498.3±1545.3	0.517
**IL-10**	51.5±186.4	39.1±87.1	0.773
**TNF-α**	20.2±13.6	35.9±38.3	0.079
**Underlying Diseases**			
**Congestive heart failure**	16 (48.5%)	8 (21.1%)	**0.015**
**Diabetic mellitus**	10 (30.3%)	6 (15.8%)	0.144
**Chronic kidney dysfunction**	6 (18.2%)	4 (10.5%)	0.355
**Solid tumor**	7 (21.2%)	10 (26.3)	0.615
**Immune system disease**	4 (12.1%)	3 (7.9%)	0.551
**Hospital Mortality (n, %)**	7 (21.2%)	9 (23.7%)	0.804

APACHE II, acute physiology and chronic health evaluation II; SOFA, sequential organ failure

assessment. WBC, white blood cell. NK cell, natural killer cell. C3, complement factor 3; C4, complement factor 4; TNF, tumor necrosis factor.

Supplement Table 2. Comparison of positive results and agreement among mNGS and CMT method in patients.

**Blood culture and Plasma mNGS**			
	CMT+	CMT-	
NGS+	31	15	46
NGS-	7	18	25
**Total**	38	43	**71**
**BALF culture and mNGS**			
	CMT+	CMT-	
NGS+	20	9	29
NGS-	0	2	2
**Total**	20	11	**31**

mNGS, metagenomic next-generation sequencing. CMT, conventional microbiological test.

Supplement Table 3. Agreement calculations between mNGS and CMT (as comparative method) on blood and BALF specimen.

Index	Blood culture and Plasma mNGS	BALF culture and mNGS
positive percent agreement, PPA	81.6% (95%CI: 66.6-90.8%)	100.0% (95% CI: 83.9-100%)
negative percent agreement, NPA	54.5% (95%CI: 38.0-70.2%)	18.2% (95% CI: 5.1-47.7%)
overall percent agreement, OPA	69.0% (95% CI: 57.5-78.6%)	71.0% (95% CI: 53.4-83.9%)

mNGS, metagenomic next-generation sequencing. CMT, conventional microbiological test. CI: confidence interval
